# The magnitude and correlates of esophageal Varices among newly diagnosed cirrhotic patients undergoing screening fibre optic endoscope before incident bleeding in North-Western Tanzania; a cross-sectional study

**DOI:** 10.1186/s12876-019-1123-9

**Published:** 2019-11-29

**Authors:** Daniel W. Gunda, Semvua B. Kilonzo, Zakhia Mamballah, Paulina M. Manyiri, David C. Majinge, Hyasinta Jaka, Benson R. Kidenya, Humphrey D. Mazigo

**Affiliations:** 1 Department of medicine, Weill Bugando School of Medicine, P.O Box 1464, Mwanza, Tanzania; 2 Department of medicine, Bugando medical center, 1370 Mwanza, Tanzania; 3 Lake Zone Health Training institute, 11351 Bugando Mwanza, Tanzania; 4 Department of Biochemistry and Molecular Biology, Weill Bugando School of Medicine, Mwanza, Tanzania; 5 Department of Parasitology, Weill Bugando School of Medicine, 1464 Mwanza, Tanzania

**Keywords:** Liver cirrhosis, Esophageal varices, Non-invasive predictors, Northwestern Tanzania

## Abstract

**Background:**

Bleeding esophageal varices is a deadly complication of liver cirrhosis. Guidelines recommend an early diagnosis of esophageal varices before incident bleeding by screening all patients diagnosed with liver cirrhosis. Though it has been reported elsewhere that the presence of esophageal varices varies widely among cirrhotic patients this has not been assessed in Tanzania since endoscopy is not readily available for routine use in our setting. This study was designed to determine the prevalence of esophageal varices and assess the utility of clinical parameters in predicting the presence of varices among cirrhotic patients in northwestern Tanzania.

**Methods:**

A cross-sectional analysis of adult patients with liver cirrhosis was done at Bugando Medical Centre. Demographic, clinical, laboratory and endoscopic data were collected and analyzed using STATA 13. The presence of esophageal varices was detected using endoscopic examination and associated factors were assessed by logistic regression. The predictive value of clinical predictors was also assessed by calculating sensitivity and specificity.

**Results:**

A total of 223 patients were enrolled, where 88 (39.5%; 95%CI: 33.0–45.9) had esophageal varices. The varices were independently associated with increased age (OR: 1.02; 95%CI: 1.0–1.04; *p* = 0.030); increased splenic diameter (OR:1.3; 95%CI:1.2–1.5; *p* <  0.001), increased portal vein diameter (OR:1.2; 95%CI: 1.07–1.4; *p* = 0.003), having ascites (OR: 3.0; 95%CI: 1.01–8.7; *p* = 0.046), and advanced liver disease (OR: 2.9; 95%CI: 1.3–6.7; *p* = 0.008). PSDR least performed in predicting varices, (AUC: 0.382; 95%CI: 0.304–0.459; cutoff: < 640; Sensitivity: 58.0%; 95%CI: 46.9–68.4; specificity: 57.0%; 95%CI: 48.2–65.5). SPD had better prediction; (AUC: 0.713; 95%CI: 0.646–0.781; cut off: > 15.2 cm**;** sensitivity: 65.9%; (95% CI: 55–75.7 and specificity:65.2%; 95%CI: 56.5–73.2), followed by PVD, (AUC: 0.6392; 95%CI: 0.566–0.712;cutoff: > 1.45 cm; sensitivity: 62.5%; 95CI: 51.5–72.6; specificity: 61.5%; 95%CI: 52.7–69.7).

**Conclusion:**

Esophageal varices were prevalent among cirrhotic patients, most of which were at risk of bleeding. The non-invasive prediction of varices was not strong enough to replace endoscopic diagnosis. However, the predictors in this study can potentially assist in the selection of patients at high risk of having varices and prioritize them for endoscopic screening and appropriate management.

## Background

Liver cirrhosis is a chronic disease of the liver which is commonly complicated by increased portal venous pressure and formation of esophageal varices [1]. The development of esophageal varices has been reported previously in up to 80% of patients with liver cirrhosis. Esophageal varices on the other hand frequently complicate into fatal upper gastrointestinal bleeding (UGIB) [2]. The risk of bleeding increases with severity of esophageal varices being highest with large varices. Additionally, the mortality associated with bleeding varices is extremely high between 20 and 35% even with the best in hospital care [3, 4]. There is also a high rate of recurrence of bleeding in up to 60% of the survivors [5, 6]. Early detection of esophageal varices and timely initiation of prophylactic treatment will potentially minimize the risk of variceal bleeding and the associated mortality [7–9].

Based on this, guidelines recommend endoscopic screening of all cirrhotic patients for esophageal varices at the time of diagnosis. However endoscopic services are still limited in most resource-limited countries (RLCs) where it is still relatively expensive for routine screening of esophageal varices and it is not readily available. Additionally, the prevalence of esophageal varices among patients with liver cirrhosis is variable and some patients who are subjected to endoscopic screening may have no varices at all. Some authors believe that doing endoscopy to all patients in a resource-limited setting may unnecessarily overburden the available resources with preventable costs [7, 10].

In this background, several studies have advocated the use of non-invasive methods to identify patients at high risk of having varices subsequently minimizing the use of endoscopies in low-risk patients [11–13]. Platelet count-to-spleen diameter ratio (PSDR) is one of the non-invasive tools recommended for this purpose [14]. This tool is simple and less expensive as reported in previous studies [14, 15]. However, there is a paucity of published data regarding the role of routine diagnostic endoscopy among cirrhotic patients before incident bleeding in Tanzania. This study was designed to determine the prevalence and risk factors of esophageal varices and assess the utility of non-invasive predictors of esophageal varices among cirrhotic patients in the North-western part of Tanzania.

## Material and methods

This was a cross-sectional study which involved all adult patients diagnosed to have liver cirrhosis at Bugando Medical Center (BMC) between January 2015 and December 2017. The study was conducted at BMC medical outpatients department. A minimum sample size of 205 was estimated from Leslie Kish formula (1965) for cross-sectional studies assuming 26% of patients had esophageal varices at diagnosis of liver cirrhosis [12] with a tolerable error of 0.06 at 95%CI. Patients suspected to have liver cirrhosis including those with jaundice; ascites and splenomegaly among others were reviewed at gastroenterology and hepatology clinic. After consent, these patients underwent a Hepato portal ultrasound (USS) scan by consultant radiologists or experienced sonographers. Those who had liver cirrhosis subsequently underwent oesophagogastroduodenoscopy (OGD) screening for esophageal varices. The OGD procedures were done by a team of gastroenterologists and experienced endoscopists in the department.

Additional tests that were done include hepatitis B and C virus (HBV and HCV) test, liver function tests (LFT); markers of liver injury (ALT & AST) and full blood picture (FBP). Any attendant complications were treated accordingly including correction of anemia, initiation of prophylactic treatment against esophageal varices including Non-selective beta-blockers (NSBB) and endoscopic variceal ligation (EVL) as per available BMC medical guideline. The patients’ data were documented and patients were serially enrolled until the desired sample size was reached.

Data were computerized using Epi data version 3.1 and STATA version 13 (Stata Corp LP, college station, TX) was used for analysis. Continuous variables were summarized as medians with interquartile range (IQR) while categorical variables were summarized as proportions with percentages. The presence of esophageal varices was calculated and those with grades 3&4 type of varices were further sub-classified as having large varices as reported previously [16]. Univariate logistic regression followed by a multivariate logistic regression model was employed to calculate the odds ratio (OR) at 95% confidence interval (CI) to assess the degree of association between different factors and the presence of esophageal varices. On the basis of previous literatures [12, 13, 17–20], and our own clinical experience we selected age, sex, alcohol use, jaundice, ascites, hepatitis status, platelet counts (PTC), hemoglobin level, serum albumin, portal vein diameter (PVD), splenic diameter (SPD), and Child-Pugh score as potential predictors of esophageal varices. Parameters including hepatic encephalopathy, serum bilirubin, serum albumin, ascites and international normalized ratio (INR) were used to calculate the Child-Pugh score as done previously [21]. All factors with a *p*-value < 0.25 on univariate logistic regression model were subsequently included in multivariate model. Factors were considered independently associated with the presence of esophageal varices if a p-value was < 0.05. We used a Hosmer-Lemeshow test and area under receiver operating characteristcs (ROC) to assess the goodness of fit of the logistic regression model.

The predictive ability of ascites, PTC, serum albumin, SPD, PVD and PSDR for the presence of varices was assessed by calculating the sensitivity and specificity as compared to endoscopy as a gold standard technique. Hanley and McNeil’s method a ROC curve was used to determine the cutoff points with the best sensitivity and specificity for continuous variables which were reported as proportions with 95%CI [22]. PSDR was calculated as a ratio of PTC (/μL) to SPD (mm) as reported previously [14].

## Results

### Baseline characteristics of study participants

A total of 223 patients with a median age of 48 [35–59] years were included in this study. Most participants, 146 (65.47%) were male. More than a half, 128 (57.40%) were married and 120 (53.81%) were peasants. The majority of these patients, 186 (83.41%) had ascites. The median Hemoglobin levels was7.2[5.2–10.0] g/dL and median platelet count was 98 [67–139]*10^3/μL. Following an assessment of the severity of liver cirrhosis by Child-Pugh classification, most patients, and 178 (79.8%) had less severe liver disease of class A/B and only about 45 (20.2%) of the study participants had severe disease of class C **(**Table 1**).**

**Table 1:** General Study Characteristics among 223 Study Participants with liver cirrhosis

Variables	Frequency	Percentage Or Medians (IQR)
Gender		
Male	146	65.47
Female	77	34.53
Age in years	223	48 [35–59]
Marital status		
Divorced	15	6.73
Married	128	57.40
Single	25	11.21
Widow	31	13.90
Other	24	10.76
Occupation		
Business	22	9.9
Fishing	16	7.2
Peasant	120	53.8
Other	65	29.1
Alcohol use		
Yes	73	32.7
No	150	67.3
Hepatitis status		
HBV Positive	50	22.4
HCV Positive	6	02.7
Negative	167	74.9
Jaundice		
Yes	87	39.0
No	136	61.0
Ascites		
Yes	186	83.4
No	37	16.6
Portal vein Diameter (cm)	91	1.4 [1.2–1.6]
Platelet count (^a^10^3)/μL	223	98 [67–139]
Hemoglobin	223	7.2 [5.2–10.0]
Child-Pugh		
Class A	15	6.7
Class B	163	73.1
Class C	45	20.2

**IQR:** interquartile range, **HB**: Hemoglobin, **HB < 10 g/dL:** moderate to severe anemia, **HBV:** Hepatitis B virus, **HCV:** Hepatitis C virus, **PSDR:** Platelet to splenic diameter ratio, PVD: Portal vein diameter,

### Prevalence and associated factors of esophageal varices among 223 participants

**Fig. 1:**
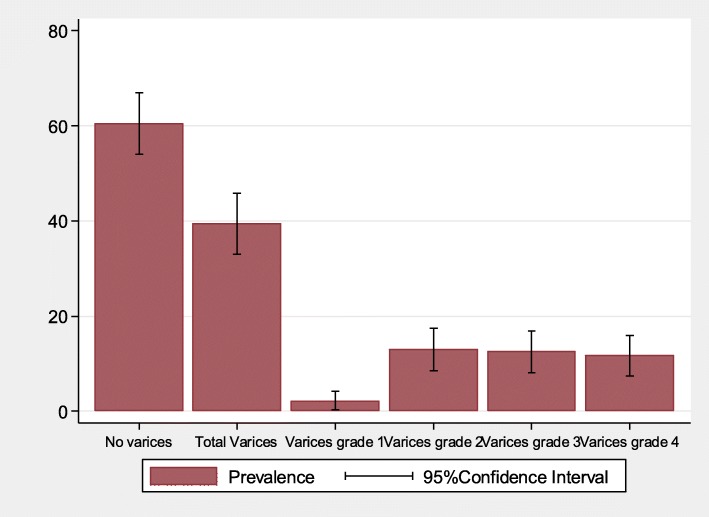
Distribution of esophageal varices among 223 participants with liver cirrhosis

**Fig. 2:**
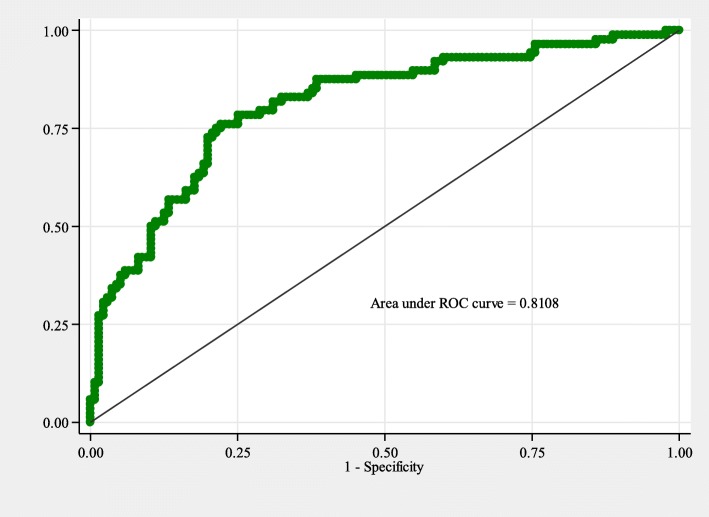
Logistic model for esophageal varices among 223 participants

Of the studied patients, 88 (39.5%; 95%CI: 33.0–45.9) were found to have esophageal varices on endoscopic examination where most of them, 54 (61.4%; 95%CI: 50.4–71.6) had large varices already **(**Fig. 1**).** On multivariate logistic regression analysis the odds of having esophageal varices were independently higher among patients with increased age (Median age of 51 vs. 45 years; OR:1.02; 95%CI: 1.0–1.04; *p* = 0.030); increased splenic diameter (Median diameter of 17 vs. 14 cm; OR:1.3; 95%C:1.2–1.5; *p* <  0.001),increased portal vein diameter (Median diameter of 15.8 vs. 14 mm; OR:1.2; 95%CI: 1.07–1.4; *p* = 0.003), having ascites (94.3% vs. 76.3%; OR: 3.0; 95%CI: 1.01–8.7; *p* = 0.046), and advanced liver disease of Child-Pugh class C, (35.2% vs. 10.3%, OR: 2.9; 95%CI: 1.3–6.7; *p* = 0.008) (Table 2). Hosmer-Lemeshow test for goodness of fit did not indicate evidence for gross lack of fit, *p* = 0.293 with the area under the ROC curve of 0.811(Fig. 2).

**Table 2:** Factors associated with esophageal varices among 223 study participants

Variables	Esophageal varices present		Unadjusted		Adjusted	
	No (*n* = 135)	Yes (*n* = 88)	OR(95%CI)	*p*-value	OR(95%CI)	*p*-value
Gender						
Female	49 (36.3)	28 (31.8)	1.0			
Male	86 (63.7)	60 (68.2)	1.2 (0.6–2.0)	0.492		
Age years	45 [34–55]	51 [37–64]	1.0 (0.9–1.1)	0.079	1.02 (1.0–1.04)	0.030
Alcohol use						
No	86 (63.7)	64 (72.7)	1.0			
Yes	49 (36.3)	24 (27.3)	0.6 (0.3–1.2)	0.162	0.9 (0.5–1.8)	0.854
Jaundice						
No	85 (63.0)	51 (57.9)	1.0			
Yes	50 (37.0)	37 (42.1)	1.2 (0.7–2.0)	0.454		
Hepatitis status						
HBV positive	30 (22.2)	20 (22.7)	1.0 (0.5–1.9)	0.930		
HCV positive	3 (2.2)	3 (3.4)	1.5 (0.3–7.8)	0.595		
Negative	100 (74.1)	65 (73.9)	1.0 (0.5–1.8)	0.972		
Spleen size (cm)	14 [13–16]	17 [15–18]	1. 5 [1.2–1.6]	< 0.001	1.3 (1.2–1.5)	< 0.001
PVD (mm)	14 [1.2–1.6]	15.8 [1.4–1.7]	1.3 [1.1–1.5]	< 0.001	1.2 (1.07–1.4)	0.003
Ascites						
No	32 (23.7)	05 (5.7)	1.0			
Yes	103 (76.3)	83 (94.3)	5.1 (1.9–13.0)	0.001	3.0 (1.02–8.7)	0.046
Hemoglobin(g/dL)	8.1 [5.7–10]	6.3 [5.2–9]	0.9 [0.7–1.0]	0.016	0.8 (0.7–1.0)	0.043
PLT (^a^10^3)/μL!	105 [72–147]	96 [62.5–129]	1.0 (0.9–1.1)	0.310		
Serum Albumin	25 [24–35]	25 [23–32]	0.97 [0.94–.0]	0.070	0.97 (0.9–1.02)	0.264
Child Pugh class C						
No	121 (89.7)	57 (64.8)	1.0			
Yes	14 (10.3)	31 (35.2)	4.7 (2.3–9.5)	< 0.001	2. 9 (1.3–6.7)	0.008

**CI:** Confidence interval, HB: Hemoglobin, **HB < 10 g/dL:** moderate to severe anemia, **HBV:** Hepatitis B virus, **HCV:** Hepatitis C virus, **PLT**: Platelet; **PSDR:** Platelet to splenic diameter ratio**, PVD:** Portal vein diameter,

### Noninvasive prediction of esophageal varices among 223 study participants

**Fig. 3:**
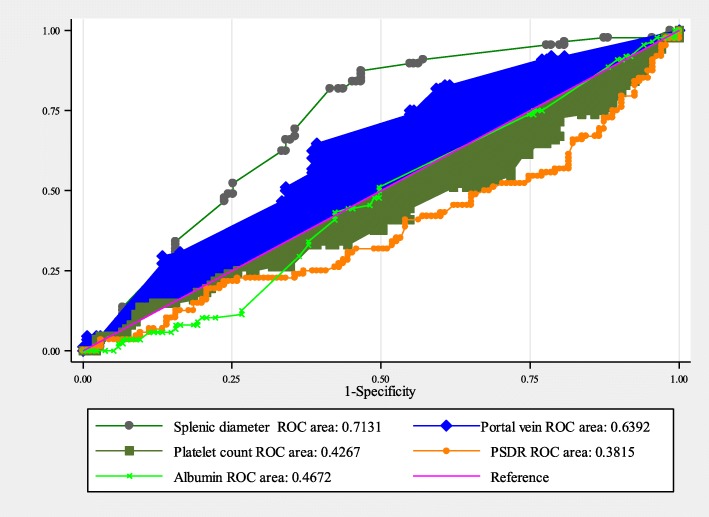
The ROC curve for non-invasive prediction of varices among 233 participants

In the current study, PSDR was found to be the least performing parameter in predicting esophageal varices, with an area under the ROC curve of 0.382 (95%CI: 0.304–0.459). The best cutoff point was < 640 with a sensitivity and specificity of 58.0% (95%CI: 46.9–68.4) and 57.0% (95%CI: 48.2–65.5) respectively. Ascites was highly sensitive, 94.3% (95%CI: 87.2–98.1), however, this parameter had a very low specificity of 23.7% (95%CI: 16.8–31.7). SPD had a fairly better performance in detecting esophageal. The area under the ROC curve was 0.713 (95%CI: 0.646–0.781); with the best cutoff point of 15.2 cm; the sensitivity and specificity was 65.9% (95% CI: 55–75.7) and 65.2% (95%CI: 56.5–73.2) respectively. The PVD had a second better prediction of varices, (AUC: 0.6392; 95%CI: 0.566–0.712; cutoff point: > 14.5 mm sensitivity: 62.5% (95CI: 51.5–72.6); specificity: 61.5% (95%CI: 52.7–69.7) (Table 3 **&** Fig. 3).

**Table 3:** Predictive values of non-invasive factors for esophageal varices among 223 participants

Variable	AUC	95%CI	SE	Cutoff point	Sensitivity	95%CI	Specificity	95%CI
SPD	0.713	0.646–0.781	0.0345	> 15.2	65.9	55.0–75.7	65.2	56.5–73.2
PVD	0.655	0.583–0.736	0.0372	> 1.45	62.5	51.5–72.6	61.5	52.7–69.7
PTC	0.427	0.348–0.506	0.0402	< 98.0	59.1	48.1–69.5	54.8	46.0–63.4
PSDR	0.382	0.304–0.459	0.0393	< 640	58.0	46.9–68.4	57.0	48.2–65.5
SALB	0.467	0.392–0.543	0.0385	< 2.57	52.3	41.3–63.0	49.8	40.9–58.3
ASCI	NA	NA	NA	NA	94.3	87.2–98.1	23.7	16.8–31.7

**ALB:** Albumin; **AUC:** area under curve; **ASCI:** Ascites; **CI:** Confidence interval; **NA**: not applicable; **PTC:** Platelet count; **PSDR**: Platelet to splenic diameter ratio; **SE**: standard error; **SPD**: Splenic Diameter;

## Discussion

The objective of this study was to determine the prevalence and associated factors of esophageal varices among patients diagnosed to have liver cirrhosis. Overall, 88 (39.5, 95%CI 33.0–45.9) of the studied patients were found to have esophageal varices where 54 (61.36%) had large varices. The presence of esophageal varices was independently associated with older age, larger portal vein diameter, larger splenic diameter, ascites, and advanced Child-Pugh classification of liver disease.

The prevalence of esophageal varices in the current study is similar to previous reports from the USA in 2007. In this study, 91 patients with primary biliary cirrhosis underwent OGD and which 34 (37.0%) were reported to have associated esophageal varices [23]. However earlier in 2004 a slightly lower prevalence of esophageal varices among 47(26.0%) participants was reported in a study of 183 patients with liver by Zein and colleagues at Mayo Clinic in the USA [12]. Comparatively higher prevalence rates of esophageal varices ranging between 51 and 91.3% were reported in several other studies [13, 17, 18] including a study from South Carolina, China, and India.

The wide difference in the prevalence of esophageal varices that we are observing could partly be due to differences in the causes of liver cirrhosis among studied patients. Most studies have reported esophageal varices among patients with specific types of liver cirrhosis including viral hepatitis and primary biliary cirrhosis among others [12, 18, 24]. As an example, it can be seen that patients with biliary cirrhosis had the lowest prevalence of esophageal varices, (26.0% vs.74.7%) as compared to those with hepatitis B related cirrhosis [12, 18]. Our current study likely included a mixed group of patients with different etiologies of liver cirrhosis. For instance, a total of 73 (32.4%) of studied patients were drinking alcohol and the other 56 (25.1%) were positive for Hepatitis B and C.

The other possible explanation for this difference in the prevalence of esophageal varices could be due to the disparity in the severity of liver disease. Prior studies have indicated that esophageal varices are commonest among patients with advanced liver cirrhosis and thus the reported prevalence is likely to be much higher in studies involving patients with advanced liver disease [24–26]. In our current study, nearly 80% of studied patients had less advanced liver disease and only about 20% had advanced liver disease that was also more likely to have esophageal varices as compared to those with less advanced liver disease (35.2% vs. 10.3%, OR = 2.9 *p* = 0.008).

Even with these differences, the clinical relevance of these findings remains practically similar. It is known that bleeding occurs in up to 35% of patients with cirrhosis without prophylaxis [4, 27, 28] with high mortality ranging between 20 and 40% in most studies [3, 4]. Our study involved patients who had never reported any overt bleeding. In a similar context, it may be inferred that about 35% of these participants may suffer fatal bleeding within 2 years with mortality that may be over 20% without initiation of appropriate primary preventive treatment.

Furthermore, cirrhotic patients without varices at diagnosis will subsequently develop varices and those with small varices will develop large varices at a rate of about 8% a year as reported by Garcia et al. [29]. In our study, about 60% had no varices on endoscopic screening and about 34 (15.2%) had small varices. This also suggests that without prophylaxis each year about 8% of our patients without esophageal varices will potentially develop esophageal varices in addition to 8% risk of developing large varices and bleeding among those with small varices. Guidelines recommend the initiation of primary prophylaxis to reduce the incidence of these unfavorable outcomes. Follow-Up endoscopy is indicated every 1–2 years among patients with small varices and those without varices [9, 30].

Endoscopy remains extremely important in diagnosis, follow up and treatment of patients varices among cirrhotic patients. Factors that can positively augment the existence of varices are potentially useful in the timing of endoscopic examination in this subgroup of patients in areas where endoscopy is not readily available for routine use. Similar to findings in our current study, increasing age was also shown to have a significant association with the presence of esophageal varices in a study by Zein et al. [12]. However, Levy et al. and Hong et al., in their studies did not find any significant statistical association between age and presence of esophageal varices in their study participants [18, 23].

Contrary to our finding several other studies have reported an independent association of thrombocytopenia with presence of esophageal varices among cirrhotic patients including an earlier study by Madhora and colleague from the USA in 2002 [13], but also reported a study by Cherian et al. from India [17] and Nada, et al. from Morocco [19]. Thrombocytopenia has been attributed to thrombopoietin deficiency in advanced liver disease and possible increased destruction of platelets due to hypersplenism among other mechanisms [31]. In our current study patients with esophageal varices only tended to have lower platelet counts, (96*10^3 vs. 105*10^3/μL, OR: 1.0; 95%CI: 0.9–1.1; *p* = 0.310), possibly because most of them had less advanced liver disease.

In the current study patient with esophageal varices were more likely to have both larger splenic size (17 vs. 14 cm, OR = 1.5, *p* <  0.001) and portal vein diameter (15.8 vs. 14 mm, OR = 1.2; 95%CI: 1.07–1.4; *p* = 0.003). association of esophageal varices with splenic and portal vein diameter were also assessed in previous studies by Cherian et al. from India [17] and Hong et al. from China [18]. In these studies larger splenic and portal vein diameters were also reported to have an independent association with the presence of esophageal varices among cirrhotic patients similar to our findings.

Also similar to our study, cirrhotic patients with ascites were shown to have an increased risk of having esophageal varices in a study from Morocco [19]. In addition to ascites patients with liver cirrhosis in Brazil were also found to have low serum albumin of less than 3.5 g/dL [20], but also Sharma et al. from India found that patients with large esophageal varices were additionally more likely to have anemia and reduced white blood cell count [32]. In our study, there was no significant difference in serum albumin levels between those with and without esophageal varices. Also though patients with esophageal varices were more likely to have more severe anemia this was negatively associated with varices suggesting possible multifactorial nature anemia.

In the current study also patients with esophageal varices were more likely to have a higher Child-Pugh score. Similar findings were reported by Cherian et al. [17]. In this study in addition to splenomegaly and thrombocytopenia liver cirrhosis of Child-Pugh B/C was reported as an independent predictor of esophageal varices. Also in 2007 an analysis of large data from clinical outpatient research initiative on use of endoscope in screening cirrhotic patients similarly indicated that esophageal varices were more commonly found among cirrhotic patients with Child-Pugh class B/C (71.9% vs.47) as compared to those in Child-Pugh class A. In agreement to our findings these patients also were indicated to be more likely to have larger esophageal varices [33].

The assessment for predictive ability of these factors found out that low platelet count< 98*10^3/μL had a sensitivity and specificity of 59.1% (95%CI: 48.1–69.5) and 54.8% (95%CI: 46.0–63.4) respectively. These predictive rates are comparatively similar to those reported in a review article by Colli et al. with sensitivity and specificity of 63–77% and 69–88% respectively however the cutoff points in this review were set at 140 and 150 involving patients with liver cirrhosis and splenic vein thrombosis [34]. With a platelet cutoff of 100cell/μL in Italy, thrombocytopenia had a much high sensitivity of 89% with a very low specificity rate of 28% in predicting esophageal varices [35]. A lower specificity rate of thrombocytopenia in predicting varices was as well reported in Greece [36].

In the current study splenomegaly better predictive ability (AUC: 0.713; 95%CI: 0.646–0.781; cutoff point: 15.2 cm; sensitivity: 65.9% (95% CI: 55–75.7); specificity: 65.2% (95%CI: 56.5–73.2). these comparatively similar to those reported by Madhotra in 2002 where splenomegaly was found to have a sensitivity and specificity of 75 and 57% respectively in predicting esophageal varices [13]. But these figures are also within ranges (sensitivity: 75–91%; specificity: 46–62%) in a recent review article by Thomopoulos and colleagues [34]. Though the performance of platelet count to splenic diameter ratio (PSDR) in our study showed a slightly lower sensitivity and specificity were, 58.0% (95%CI, 46.9–68.4) and 57.0% (95%CI, 48.2–65.5) respectively as compared to studies by Zamil et al. [37], at cutoff values of 909 (897–921), most studies had reported similar results with sensitivity of 72–93% and specificity of 52–77% [34]. Use of PVD (AUC: 0.6392; 95%CI: 0.566–0.712, cutoff point at > 14.5 mm) had a sensitivity of 62.5% (95CI: 51.5–72.6) and specificity, 61.5% (95%CI: 52.7–69.7). These findings are similar to those reported by Jamil et al. with sensitivity of 51.25% (95%CI: 39.8–62.6) and specificity of 65.71% (95%CI: 53.4–76.7) with comparable AUC: 0.591, and a lower cutoff point of 12 mm [37]. High predictive values of the PVD for varices was reported in Nepal at a cutoff point of 12.25 mm (sensitivity: 92.72%; specificity: 90%) [38].

Ascites has a high sensitivity, 94.3% (95%CI: 87.2–98.1); however with very low specificity, 23.7% (95%CI: 16.8–31.7). These findings are similar to those reported by Thomopoulos et al. (sensitivity: 95%; specificity: 37% [36]. Similarly, earlier in 1999 another study reported that ascites had a higher sensitivity of 100% in predicting esophageal varices however with a low specificity of 51% [39]. Compared to findings from a study by Zein and colleagues, low serum albumin levels in our study had similar sensitivity (52.3%; (95%CI: 41.3–63.0) vs. 52%) and a slightly lower specificity, (49.8%; (95%CI: 40.9–58.3) vs.69%) in predicting esophageal varices [12]. But also Khan and colleagues a similar sensitivity of 53.25% to our study with a much higher specificity of 91% [40].

This study is liable to some limitations. Being a single-center study the results from this study may not be generalizable. But also the cross-sectional nature of this study limits the understanding and assessment of the temporal sequence of events in this subgroup of patients. However, this is the first study assessing the prevalence of esophageal varices and its predictors among newly diagnosed cirrhotic patients before overt bleeding in Tanzania where endoscopic services are still scarce and readily expensive for routine use.

## Conclusions

In conclusion, this study shows that esophageal varices are prevalent among patients diagnosed with liver cirrhosis, with a predominance of large varices that are at risk of bleeding. The performance of most non-invasive parameters can’t replace the paramount importance of endoscopy among patients who are newly diagnosed with liver cirrhosis. Although a large proportion of patients did not have varices at diagnosis of cirrhosis, the predictors identified in this study could significantly augment the selection and prioritization of patients who might need immediate scoping. Patients with increased age, increased portal vein diameter, increased splenic diameter, ascites and advanced liver disease by Child-Pugh score are more likely to have esophageal varices and thus can benefit from prioritized endoscopic examination and appropriated primary prophylaxis.

## Data Availability

We declare that the supporting data can be available upon request from the corresponding author.

## Abbreviations

ALT: Alanine aminotransferase
AST: Aspartate aminotransferase
AUC: Area under the Curve
BMC: Bugando Medical centre
CI: Confidence interval
CUHAS: Catholic university of health and allied sciences
EVL: Endosco11pic band ligation
FBP: Full Blood Picture
HBV: Hepatitis B virus
HCV: Hepatitis C virus
INR: International normalized ratio
IQR: Interquatile range
LFT: Liver function test
NSBB: Non selective B blockers
OGD: Oesophago gastro duodenoscopy
OR: Odds ratio
PSDR: Platelet count to splenic diameter ratio
PTC: Platelet count; PVD: Portal vein diameter
RLCs: Resource limited countries
ROC: Receiver operating curve
SPD: Splenic diameter
UGIB: Upper gastro intestinal bleeding
USA: United states of America
USS: Ultrasound

## References

[1] de Franchis R, Primignani M (2001). Natural history of portal hypertension in patients with cirrhosis. Clin Liver Dis.

[2] D'Amico G, Morabito A (2004). Noninvasive markers of esophageal varices: another round, not the last. Hepatology.

[3] Graham DY, Smith JL (1981). The course of patients after variceal hemorrhage. Gastroenterology.

[4] D'Amico G, Garcia-Tsao G, Pagliaro L (2006). Natural history and prognostic indicators of survival in cirrhosis: a systematic review of 118 studies. J Hepatol.

[5] D'Amico G, De Franchis R (2003). Upper digestive bleeding in cirrhosis. Post-therapeutic outcome and prognostic indicators. Hepatology.

[6] Moledina SM, Komba E (2017). Risk factors for mortality among patients admitted with upper gastrointestinal bleeding at a tertiary hospital: a prospective cohort study. BMC Gastroenterol.

[7] Spiegel BM (2003). Endoscopic screening for esophageal varices in cirrhosis: is it ever cost-effective?. Hepatology.

[8] Aoki N (2000). Decision analysis of prophylactic treatment for patients with high-risk esophageal varices. Gastrointest Endosc.

[9] Garcia-Tsao G (2007). Prevention and management of gastroesophageal varices and variceal hemorrhage in cirrhosis. Hepatology.

[10] Di Pascoli L (2014). Cost-effectiveness analysis of beta-blockers vs endoscopic surveillance in patients with cirrhosis and small varices. World J Gastroenterol.

[11] Zaman A (2001). Risk factors for the presence of varices in cirrhotic patients without a history of variceal hemorrhage. Arch Intern Med.

[12] Zein CO, Lindor KD, Angulo P (2004). Prevalence and predictors of esophageal varices in patients with primary sclerosing cholangitis. Hepatology.

[13] Madhotra R (2002). Prediction of esophageal varices in patients with cirrhosis. J Clin Gastroenterol.

[14] Giannini E (2003). Platelet count/spleen diameter ratio: proposal and validation of a non-invasive parameter to predict the presence of oesophageal varices in patients with liver cirrhosis. Gut.

[15] Ying L (2012). Performance of platelet count/spleen diameter ratio for diagnosis of esophageal varices in cirrhosis: a meta-analysis. Dig Dis Sci.

[16] Abby Philips C, Sahney A (2016). Oesophageal and gastric varices: historical aspects,classification, and grading: everything in one place. Gastroenterol Rep (Oxf).

[17] Cherian JV (2011). Non-invasive predictors of esophageal varices. Saudi J Gastroenterol.

[18] Hong WD (2009). Predictors of esophageal varices in patients with HBV-related cirrhosis: a retrospective study. BMC Gastroenterol.

[19] Nada L (2015). Noninvasive predictors of presence and grade of esophageal varices in viral cirrhotic patients. Pan Afr Med J.

[20] Fagundes ED (2008). Clinical and laboratory predictors of esophageal varices in children and adolescents with portal hypertension syndrome. J Pediatr Gastroenterol Nutr.

[21] Planas R (2004). Natural history of decompensated hepatitis C virus-related cirrhosis. A study of 200 patients. J Hepatol.

[22] Hanley JA, McNeil BJ (1982). The meaning and use of the area under a receiver operating characteristic (ROC) curve. Radiology.

[23] Levy C (2007). Prevalence and predictors of esophageal varices in patients with primary biliary cirrhosis. Clin Gastroenterol Hepatol.

[24] Zaman A (1999). Factors predicting the presence of esophageal or gastric varices in patients with advanced liver disease. Am J Gastroenterol.

[25] Merli M (2003). Incidence and natural history of small esophageal varices in cirrhotic patients. J Hepatol.

[26] Bosch J (2008). The management of portal hypertension: rational basis, available treatments, and future options. J Hepatol.

[27] Poynard T (1991). Beta-adrenergic-antagonist drugs in the prevention of gastrointestinal bleeding in patients with cirrhosis and esophageal varices. An analysis of data and prognostic factors in 589 patients from four randomized clinical trials. Franco-Italian multicenter study group. N Engl J Med.

[28] Kraja B (2017). Predictors of esophageal varices and first variceal bleeding in liver cirrhosis patients. World J Gastroenterol.

[29] Garcia-Tsao G (2007). Prevention and management of gastroesophageal varices and variceal hemorrhage in cirrhosis. Am J Gastroenterol.

[30] Tripathi D (2015). U.K. guidelines on the management of variceal hemorrhage in cirrhotic patients. Gut.

[31] Mitchell O (2016). The pathophysiology of thrombocytopenia in chronic liver disease. Hepat Med.

[32] Sharma SK, Aggarwal R (2007). Prediction of large esophageal varices in patients with cirrhosis of the liver using clinical, laboratory and imaging parameters. J Gastroenterol Hepatol.

[33] Kovalak M (2007). Endoscopic screening for varices in cirrhotic patients: data from a national endoscopic database. Gastrointest Endosc.

[34] Colli A (2017). Platelet count, spleen length, and platelet count-to-spleen length ratio for the diagnosis of oesophageal varices in people with chronic liver disease or portal vein thrombosis. Cochrane Database Syst Rev.

[35] Schepis F (2001). Which patients with cirrhosis should undergo endoscopic screening for esophageal varices detection?. Hepatology.

[36] Thomopoulos KC (2003). Non-invasive predictors of the presence of large oesophageal varices in patients with cirrhosis. Dig Liver Dis.

[37] Jamil Z, Malik M, Durrani AA (2017). Platelet count to splenic diameter ratio and other noninvasive markers as predictors of esophageal varices in patients with liver cirrhosis. Turk J Gastroenterol.

[38] Bhattarai S (2017). Non-invasive predictors of gastro-Oesophageal Varices. JNMA J Nepal Med Assoc.

[39] Ng FH (1999). Prediction of oesophagogastric varices in patients with liver cirrhosis. J Gastroenterol Hepatol.

[40] Khan H (2009). A marker of esophageal varices in chronic liver disease due to hepatitis B and C. Rawal Med J.

